# Methionine Supplementation during Pregnancy of Goats Improves Kids’ Birth Weight, Body Mass Index, and Postnatal Growth Pattern

**DOI:** 10.3390/biology11071065

**Published:** 2022-07-18

**Authors:** Diego Castillo-Gutierrez, Luisa E. S. Hernández-Arteaga, Manuel J. Flores-Najera, Venancio Cuevas-Reyes, Juan M. Vázquez-García, Catarina Loredo-Osti, Sergio Beltrán-López, Gilberto Ballesteros-Rodea, Antonio Gonzalez-Bulnes, Cesar A. Meza-Herrera, Cesar A. Rosales-Nieto

**Affiliations:** 1 Facultad de Agronomía y Veterinaria, Universidad Autónoma de San Luis Potosí, San Luis Potosí 78321, Mexico; a252939@alumnos.uaslp.mx (D.C.-G.); socorro.hernandez@uaslp.mx (L.E.S.H.-A.); manuel.vazquez@uaslp.mx (J.M.V.-G.); catarina.loredo@uaslp.mx (C.L.-O.); gilberto.ballesteros@uaslp.mx (G.B.-R.); 2 Instituto Nacional de Investigaciones Forestales, Agrícolas y Pecuarias, Campo Experimental La Laguna, Matamoros 27440, Mexico; flores.manuel@inifap.gob.mx; 3 Instituto Nacional de Investigaciones Forestales, Agrícolas y Pecuarias, Campo Experimental Valle de México, Texcoco 56250, Mexico; cuevas.venancio@inifap.gob.mx; 4 Instituto Nacional de Investigaciones Forestales, Agrícolas y Pecuarias, Campo Experimental San Luis, San Luis Potosí 78431, Mexico; beltran.sergio@inifap.gob.mx; 5 Departamento de Producción y Sanidad Animal, Facultad de Veterinaria, Universidad Cardenal Herrera-CEU, CEU Universities, 46115 Alfara del Patriarca, Spain; antonio.gonzalezbulnes@uchceu.es; 6 Unidad Regional Universitaria de Zonas Áridas, Universidad Autónoma Chapingo, Texcoco 35230, Mexico; cmezah@chapingo.mx

**Keywords:** herbal methionine, late gestation, progeny performance, milk components

## Abstract

**Simple Summary:**

Methionine is an essential amino acid that contributes to protein formation, fetal development, and milk synthesis. Thus, we hypothesized that a supplementation with Optimethione^®^ during the last third of gestation in female goats will increase the maternal body live weight, the milk yield and milk composition and the offspring weight and body mass index at birth and postnatal performance. We fed (*n* = 30) or not (*n* = 30) herbal methionine Optimethione^®^ to pregnant multiparous Alpine goats from gestational day 100 until delivery. We evaluated the productive variables from the dams and offspring. Maternal intake of herbal methionine Optimethione^®^ during pregnancy did not increase the live weight or increase the milk yield and composition. However, the tested offspring variables were influenced by the maternal intake of herbal methionine Optimethione^®^ during gestation by improving the birth weight, body mass index, and postnatal growth pattern. An increase in birth weight would be expected to increase neonatal survival up to weaning. Moreover, a relative fatness is required for reproductive success; thus, offspring that are born heavier and bigger can display a faster postnatal growth and accelerate the onset of puberty and increase reproductive success.

**Abstract:**

The last third of gestation is a period of high energy and protein demand for the dam to support fetal growth and the following onset of lactation. Methionine is an essential amino acid that contributes to protein formation, fetal development, and milk synthesis; thus, is likely to have positive effects on the weight and size of the newborn and, afterward, milk yield and milk composition, which may improve growth patterns of the progeny. To test these hypotheses, we used 60 pregnant multiparous Alpine goats with similar live weights and gestational ages (~Day 100 of pregnancy; Mean ± SD; 1410 ± 14 days old and 50.4 ± 6.6 kg) and were separated into two groups: control and supplemented with the delivery. Treatments were T-MET (*n* = 30; received 1% herbal methionine Optimethione^®^ dry matter based on from Day 100 of the pregnancy to delivery) or T-CTL (*n* = 30; served as the control and did not receive methionine). The methionine powder provided individual supplementation and was adjusted every week as the live weight and dry matter intake changed. At birth, the weight, body mass index (BMI), birth type, and sex of the kids were determined. Subsequently, the progeny was weighed weekly up to weaning. Two weeks after parturition, the milk composition was recorded weekly, and the milk yield was recorded monthly. The maternal live weight at the start (Mean ± SEM; T-CTL: 50.5 ± 1.1 vs. T-MET: 50.3 ± 1.3 kg) and end (T-CTL: 54.2 ± 1.3 vs. T-MET: 52.8 ± 1.4 kg) of the experiment did not differ statistically among treatments (*p* > 0.05); however, daily live weight changes tended to differ between groups (T-CTL: 73 ± 10 vs. T-MET: 51 ± 7 g day^−1^; *p* = 0.06). The birth weight (T-CTL: 3.1 ± 0.1 vs. T-MET: 3.5 ± 0.1 kg; *p* < 0.001), daily live weight change (T-CTL: 121 ± 6 vs. T-MET: 141 ± 6 g day^−1^; *p* < 0.01), and weaning weight (T-CTL: 8.3 ± 0.2 vs. T-MET: 9.3 ± 0.3 kg; *p* < 0.01) differed between treatments. The BMI at birth (T-CTL: 0.28 ± 0.01 vs. T-MET: 0.3 ± 0.01 units kg m^−2^; *p* < 0.01) and at weaning (T-CTL: 0.85 ± 0.1 kg vs. T-MET: 1.00 ± 0.06 units kg m^−2^; *p* < 0.05) differed between treatments. Milk components (protein, fat, lactose, and solids non-fat) and milk yield were similar between treatments (*p* > 0.05). It is concluded that the inclusion of methionine in the maternal goat diet during the last third of gestation increases the birth and growth variables of the progeny but without significant influence on the milk yield and composition.

## 1. Introduction

Around 75% of the fetoplacental unit growth occurs during the last third of gestation, resulting in a reduced maternal rumen capacity that decreases the feed intake [1,2]. During this stage, the dam shifts from a non-lactating lipogenic stage to a state of high energy demand to support body maintenance and homeostasis, the growth of fetuses, and the onset of lactogenesis [3,4]. Fetal growth requires acceptable levels of maternal protein intake to support fetal tissue development (12.5% CP) [5,6,7]. Therefore, maternal intake of protein and energy during the last third of gestation need to be well-balanced; otherwise, it will jeopardize the fetal and postnatal development.

If the maternal diet does not meet the nutritional requirements, the dam will experience a catabolic state. Maternal catabolic state as a consequence of sub-nutritional results in reduced fetal development by decreasing the nutrients exchange between mother and fetus, affecting the oxygen levels, circulating concentration of metabolites, metabolic hormones, and growth factors in the fetus [8,9]. The fetus is sensitive to maternal nutrition and adapts to nutritional challenges by modulating its growth; however, the maternal catabolic state may jeopardize fetal myogenesis and adipogenesis [1]. As a result, the birth weight and vigor of the neonate are decreased and negatively affect the mother-young bonding [7,10,11]. Simultaneously, the yield and composition of colostrum and, afterward milk are negatively influenced by the maternal catabolic state [12,13]. Both reduced the colostrum yield and composition and offspring reduced birth weight can increase the mortality rate [14,15]. Offspring that are born small for gestational age display slow postnatal growth, which results in delayed puberty onset and decreased reproductive success [16,17].

In extensive production systems, as in most animal production systems in arid and semiarid regions of the world, the nutritional status of grazing animals is greatly influenced by seasonal factors [18,19]. In Northern México, female goats are sexually active during the fall and winter whilst showing seasonal anestrus during the spring and summer [20,21]. However, the nutritive value of pasture during the fall and winter may be low, which, being concomitant to the breeding period and gestation, may affect the reproductive efficiency of the females [22]. In our quest to identify low-cost alternatives, different approaches have been tested to overcome this situation, such as the use of ball moss [23] or the inclusion of *Opuntia* spp. [24,25] in animal diet. Nevertheless, there is a clear need for a low-cost alternative feed supplement that can improve the production efficiency of animals reared under these difficult conditions.

Methionine supplementation can overcome this difficult situation; whether only during late gestation or lactation or both periods is yet to be defined. Amino acids are important for the metabolism, growth, development, and immunity of organisms, and methionine is an essential amino acid and a component of all proteins [26,27]. Essential amino acids must be provided in the diet as the animal cannot synthesize them or it does but at a low rate [28]. Methionine is essential for fetal gene expression involved in metabolism, growth, and health, as it has been implied to be vital in DNA synthesis and methylation of DNA gene expression [29,30]. In addition, methionine is related to creatine production, which is an essential compound for energy production and muscle development [31]. Therefore, methionine supplementation during gestation results in increased fetal development and birth weight in different species [32,33,34,35,36]. In addition, methionine supplementation during late gestation plays an important role in the onset of lactogenesis [33,37], and during early lactation, it can increase milk yield and milk protein in dairy cows [38].

We propose to include in the maternal diet during the last third of gestation an herbal component of methionine (Optimethione^®^) with natural stability that contains L-methionine and S-adenosylmethionine [39]. Natural ingredients are desired to be included in production systems as part of the strategies to improve the productive and reproductive performance of the animals under a clean, green, and ethical production system [40]. Therefore, we hypothesized that supplementation with Optimethione^®^ during the last third of gestation in female goats will increase (1) the maternal live weight, (2) the milk yield and milk composition, and (3) the offspring weight and body mass index at birth and postnatal performance.

## 2. Materials and Methods

### 2.1. Study Site

The study was conducted at the Agronomy and Veterinary Faculty of the Autonomous University of San Luis Potosi (22°13′ N, 100°51′ W) and was approved by the Institutional Animal Care and Use Committee of the University (C19-FAI-05-86.86, 511-6/2019.-8024; 511-6/2019.-12305). All animal procedures were consistent with international guidelines [41] and with the national guidelines [42] for the care and use of laboratory animals. At this location, climate is considered desert dry and cold BsKw (wi), according to Köppen. The average annual temperature is 18 °C and with a precipitation of about 341 mm per year. The driest month is March (average of 6 mm), and most precipitation falls in June (average of 67 mm) [43]. The experiment started in August and finished in August of the following year.

### 2.2. Experimental Design

**Figure 1:**
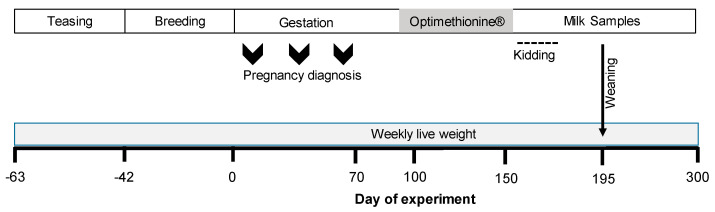
Scheme describing the experimental design that considered including herbal methionine (Optimethione^®^) in the maternal diet on the estimated gestational date 100 up to parturition. After parturition, milk yield was measured monthly and milk components (fat, protein, lactose, solids non-fat) were determined weekly.

The experimental protocol is shown in Figure 1. A total of 60 adult pregnant Alpine goats (on average 1410 ± 14 d old and 50.4 ± 6.6 kg) from the in-house flock—and afterward, their progeny—were used to investigate the effects of the supplementation with Optimethionine^®^ during the last third of gestation on maternal live weight change, offspring size (Body Mass Index: BMI), weight at birth, postnatal daily live weight gain, weaning weight, milk yield, and milk composition.

Goats were teased before the mating period for 21 days (−63 d, August). Afterward, goat pregnancy was achieved by natural mating with experienced bucks for 42 days (two full reproductive cycles, −42 d, mostly during September). Pregnancy, number of fetuses, and gestational age were assessed three times between 30 and 45 d after starting natural mating by transabdominal ultrasonography (Samsung Medison SA-600 fitted to a 4 MHz convex probe; Samsung Co. Seoul, South Korea). Pregnancy was confirmed, and gestational age was estimated by assessing the uterine depth (in early pregnancy), fetal length from crown to rump, biparietal diameter, and calcification of the fetal ribs and skull [44].

Around 95% of the goats were found to be pregnant during the first reproductive cycle. Hence, a total of 60 pregnant goats were selected on an estimated gestational day of 100 ± 0.5. These goats were randomly allocated in two pens, one for each treatment, ensuring the average live weight and estimated gestational age of the groups were similar. The treatments were 0.0% (T-CTL; Control, 100 d) and 1.0% of herbal methionine supplement Optimethionine^®^ (T-MET; dry matter based) in maternal diet.

### 2.3. Nutritional Diet and Herbal Methionine (Optimethionine^®^) Supplementation

In this study, a total mixed ration was provided in a feeder with sufficient space (30 cm of linear space per goat) to minimize competition, allowing each animal free access to consume its feed allocation. During the experiment, the last third of gestation, the diets were based on maize silage, alfalfa hay and oats hay, and fed twice daily (half of the total ration on each occasion) with the diet depicted in Table 1. The treated groups were fed once daily with herbal methionine supplementation from the estimated gestational day 100 ± 0.5 up to parturition. The diet provided for all the treatments (T-CTL and T-MET) covered the maintenance requirements with limited physical activity but not additional requirements for late pregnancy (1.35 kg dry matter, 1.76 Mcal of metabolizable energy, and 69 g of crude protein) [45], reflecting the on-practice situation for goats in semiarid regions of Northern Mexico (i.e., undernutrition during pregnancy). The nutritional diet was set to reflect the real-world situation for goats in the arid and semiarid regions of Mexico and elsewhere [18,19]. Afterward, the goats received a diet that met the nutritional requirements for lactation (1.65 kg dry matter, 2.97 Mcal of metabolizable energy, and 133 g of crude protein) [45]. Drinking water was provided ad libitum all throughout.

Herbal methionine supplement powder (Optimethionine^®^) contains *Trigonella foenumgraecum* and *Allium sativa*, providing 2.8% of natural conjugated methionine [46]. The amount of herbal methionine supplement (Optimethionine^®^) was calculated based on the daily dry matter intake of each goat (3% based on live weight). Herbal methionine supplement powder (Optimethionine^®^) was provided daily with 50 g of balanced feed (Nu-3^®^ Ganado Lechero, 18%CP) in the milking parlor of the goat unit of the faculty to ensure each female gets the right amount of methionine. On the previous day, the amount of Optimethionine^®^ (~ 13.5 g/goat/d) was mixed with the balanced feed and kept in individual paper bags. The control treatment (T-CTL) did not receive an herbal methionine supplement but 50 g of balanced feed. Herbal methionine (Optimethionine^®^) supplementation was adjusted every week as per live weight to provide the dry matter based on live weight changes and dry matter intake of each female and according to the treatment.

### 2.4. Maternal Outcomes

Goats were weighed every week from estimated gestational age 100 ± 0.5 to parturition. The data helped to determine the daily maternal live weight change during the supplementation period. To determine the impact of herbal methionine supplementation during the last third of gestation on maternal body live weight change and fetal growth, the total birth weight, the total fetal gain, and the total maternal gain were calculated. The total birth weight was calculated as the birth weight from singletons or the sum of the birth weights of twins. The total fetal gain was calculated as the total birth weight divided by 50 days. The total maternal gain was calculated by extracting the total fetal gain and the maternal live weight change.

### 2.5. Milk Yield and Composition

Milk composition was recorded weekly and milk yield was recorded monthly from two weeks after parturition (165 d, March) to 4 months later (300 d, August) (Figure 1). Once a week and while the goats were in the milking parlor, a milk sample (10 mL) from each goat was obtained and immediately analyzed for milk protein, fat, lactose, and solids-non-fat (SNF) using a Milko Tester LTD (MasterEco, Belovo, Bulgaria) after calibration for goat milk according to the manufacturer. Once a month, the milk yield was calculated by the oxytocin protocol [47]. In brief, while nurturing, goats were separated from their kids the day before milk recording, penned separately, and then hand-milked. To elicit milk letdown, goats received an intramuscular injection of 3 mL of oxytocin (20 IU mg^−1^, PiSA Agropecuaria, Hidalgo, Mexico). After five minutes, goats were milked, and the time of the first milking was recorded. Goats were re-milked approximately 3 h later, in the same order as the initial milking, following the same oxytocin protocol. Milk weight collected at the second milking and the time between milkings were recorded to obtain an estimate of milk yield per day.

### 2.6. Newborn Outcomes

Sex, birth weight, and birth type were recorded at birth. To assess offspring growth, live weights were recorded weekly from birth until weaning at 45 days old (195 d, Figure 1). In addition, the morphometric measures of the progeny (body length from rump to shoulder; body height from the ground up to the withers) were recorded at birth, and weaning [48,49]. Morphometric measures were used to determine body mass index (BMI) as an indicator of body growth and development and calculated using the following equation: BMI: ((live weight (kg)/withers height (m)/body length (m)) × 10)/100), as previously described by Tanaka et al. [48] but with a modification, as reported by Rosales-Nieto et al. [49]. Dams and progeny from each treatment were kept together during lactation.

### 2.7. Data Analyses

All data analyses were performed using the SAS statistical package SAS version 9.3 [50]. Each animal was considered an experimental unit. The experiment was organized in a completely randomized design. A Shapiro–Wilk normality test was used to show that the data were normally distributed (*p* = 0.97; PROC-UNIVARIATE).

Maternal live weight at the start of the supplementation period, maternal live weight change during the supplementation period, total birth weight, total fetal daily gain during supplementation, and total maternal gain were analyzed using linear mixed model procedures (PROC-MIXED). The fixed effect in the model was the treatment. The relationship between maternal live weight and time across the experiment was computed using mixed models (PROC-MIXED), allowing for repeated measurement. The random effect was goat ID within treatment.

Birth weight, live weight gains, weaning weight, birth BMI, and weaning BMI of the progeny were analyzed using linear mixed model procedures (PROC-MIXED). Fixed effects in the model were treatment, birth type, and progeny sex. Birth weight, live weight gains, and weaning data were included as covariates as appropriate. The maternal live weight change and kid live weight gain were fitted in a linear regression model of weight on time for each, and estimates of the regression coefficients were obtained as a measure of change by unit time.

The milk yield (at 24 h) and milk components (milk fat, milk protein, milk lactose, and solids non-fat) data were analyzed using a mixed linear model (PROC MIXED of SAS). The fixed effect in the model was the treatment. Maternal live weight, progeny sex, and birth type of the kids were included independently as covariates. The sampling date was included as a repeated measurement. The random effect was goat ID within treatment.

All two-way interactions among the fixed effects and covariates were included in each model and nonsignificant (*p* > 0.05) interactions were removed from the model. Significant differences among means for treatments within variables were analyzed using LS-MEANS of PROC MIXED.

## 3. Results

### 3.1. Maternal Live Weight Change

**Figure 2:**
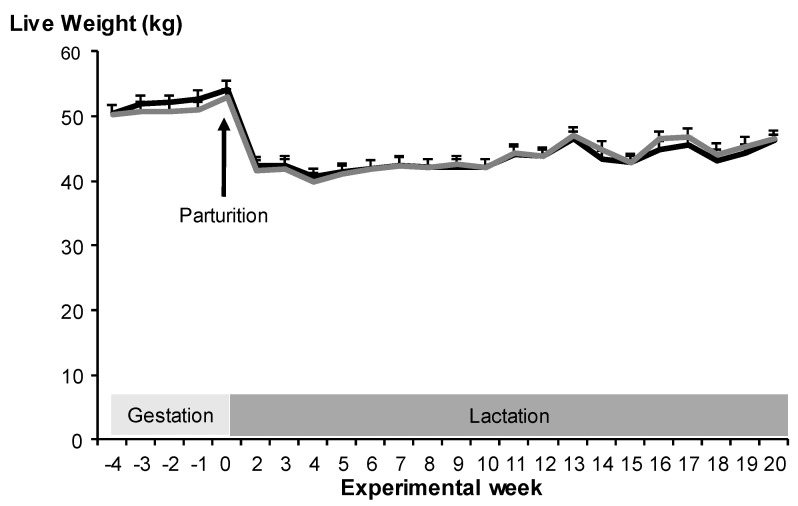
Mean maternal live weight (± SEM) of Alpine goats during late pregnancy and lactation that received herbal methionine supplement (Optimethionine^®^) at 1% (dry matter basis; grey line) or not (black line) from estimated gestational d 100 up to parturition. The interaction between treatment and sampling date was not significant (*p* = 0.99).

At enrollment (estimated gestational day 100 ± 0.5), maternal live weight was similar between treatments (Table 2; *p* > 0.05). The maternal live weight change during the last third of gestation tended to differ between treatments (Table 2; *p* = 0.06). The total birth weight, total fetal daily gain, and total maternal gain during the last third of gestation did not differ between treatments (*p* > 0.05; Table 2). Figure 2 represents the maternal live weight during lactation and similarly did not differ between treatments (*p* > 0.05) but differed across time (*p* < 0.001). The interaction between treatment and sampling date was not significant (*p* > 0.05).

### 3.2. Milk Yield and Chemical Composition

#### 3.2.1. Milk Yield

**Figure 3:**
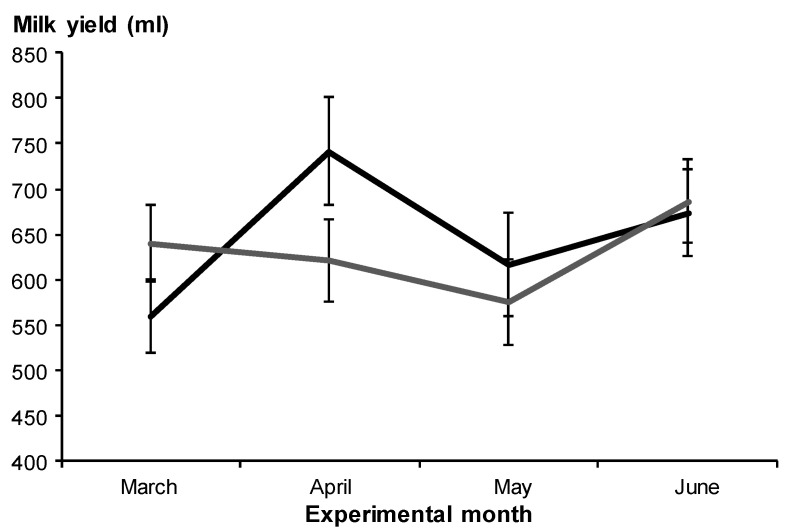
Monthly milk yield (± SEM) in Alpine goats that received herbal methionine supplement (Optimethionine^®^) at 1% (dry matter basis; grey line) or not (black line) during the last third of gestation. The interaction between treatment and sampling date was not significant (*p* = 0.20).

On average, milk yield was similar between treatments (T-CTL: 647 ± 52 mL/day vs. T-MET: 630 ± 52 mL/day; *p* > 0.05; Figure 3). Milk yield was positively influenced by sampling date (*p* < 0.05; Figure 3); but not by maternal live weight, sex, and birth type of the kids (*p* > 0.05). Time of sampling did not differ among sampling dates (*p* > 0.05). The interaction between treatment and sampling date was not significant (*p* > 0.05).

#### 3.2.2. Milk Composition

**Figure 4:**
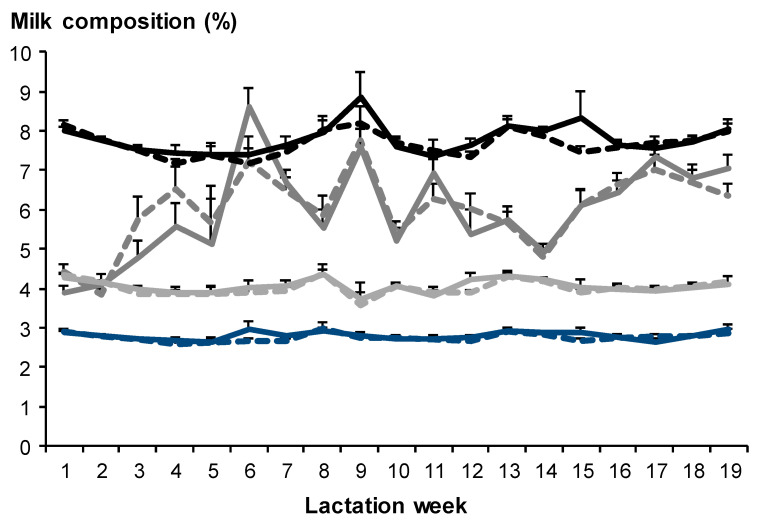
Milk fat (black line ± SEM), protein (dark grey line ± SEM), lactose (light grey line ± SEM), and solids non-fat (SNF; blue line ± SEM) from Alpine goats that received herbal methionine supplement (Optimethionine^®^) at 1% (dry matter basis; dashed line) or not (solid line) during the last third of gestation. The interaction between treatment and sampling date was not significant for Fat (*p* = 0.20), Protein (*p* = 0.40), Lactose (*p* = 0.99), and SNF (*p* = 0.70).

Across the experiment, milk fat (T-CTL: 6.0 ± 0.41% vs. T-MET: 6.0 ± 0.38%), milk protein (T-CTL: 2.8 ± 0.08% vs. T-MET: 2.8 ± 0.05%), milk lactose content (T-CTL: 4.1 ± 0.13% vs. T-MET: 4.0 ± 0.10%), and solids non-fat (SNF; T-CTL: 7.8 ± 0.23% vs. T-MET: 7.7 ± 0.16%) did not differ between T-CTL and T-MET goats (*p* > 0.05; Figure 4) but differed among sampling dates (*p* < 0.05; Figure 4). For all the variables, time of sampling differed among sampling dates (*p* < 0.001). The interaction between treatment and sampling date was not significant (*p* > 0.05).

Milk fat, milk protein, milk lactose content, and SNF did not differ between goats suckling male or female kids or goats bearing singletons or twins (*p* > 0.05). The maternal live weight was negatively associated with milk fat content (*p* < 0.001). The milk fat content decreased by 0.5% as the maternal live weight increased by 10 kg. The maternal live weight was not associated with the milk protein or milk lactose content or SNF (*p* > 0.05).

### 3.3. Offspring Outcomes

On average, when all the animals from all the treatments were considered for the analyses, male kids were heavier at birth than female kids (*p* < 0.01) and grew 18% (*p* < 0.01) faster, and were 8% heavier at weaning (*p* < 0.05). Single-born kids were heavier at birth than twin-born kids (*p* < 0.0001) and grew 37% faster (*p* < 0.0001) and were 24% heavier at weaning than twin-born kids (*p* < 0.0001).

Late-pregnancy herbal methionine supplementation was related to increases in the birth weight and size (*p* < 0.001 and *p* < 0.01, respectively), the daily live weight gains (*p* < 0.01), and the weaning weight and size (*p* < 0.01 and *p* < 0.05, respectively) (Table 3). Kids from T-MET grew 17% faster and were 12% heavier at weaning than kids from T-CTL (Table 3).

## 4. Discussion

The hypotheses were that the inclusion of herbal methionine in the maternal diet during the last third of gestation may result in increased maternal live weight and, as consequence, increasing weight and size at birth and inducing faster postnatal development in the progeny. The results indicate that, although the maternal consumption of methionine did not positively impact maternal live weight changes, it increased the weight and BMI at birth of the progeny and accelerated the postnatal growth without changes in milk yields and composition. Hence, it can be extrapolated that, in conditions where food allowance is reduced, supplementation at 1% of dry matter based on herbal methionine can overcome this difficult situation.

The changes in the maternal live weight showed a trend to differ between treatments; however, there were no differences in such weight at the end of the experiment. Goats are very tolerant animals and under conditions where the food conditions are scarce, they can adapt their metabolism and still reproduce [20,23,51,52]. After analyzing the fetal growth and dam live weight changes, we concluded that, despite the increased live weight towards the end of the experiment, the goats used their body reserves to compensate for the nutritional deficiency in the diet [20,53]. Moreover, the increased maternal live weight was due to the fetal growth and development [1], which as we will explain below, it affected the birth weight outcome. This confirms that the diet met our objectives as the amount of food offered during late gestation did not meet the nutritional requirements for a pregnant goat.

We were expecting that herbal methionine supplementation would compensate for the live weight loss and increases the maternal live weight, as amino acids are known to be anabolic factors that stimulate protein synthesis, which is necessary for tissue growth [54,55]. Our results are in agreement with those of Liu et al. [56], as these authors did not observe a difference in maternal live weight after supplementing 6.3 g/d of methionine for 60 d in late gestation in Merino sheep. Perhaps, other factors are more important toward parturition as nutritional and hormonal factors may have contributed to the lack of response in the live weight of goats by the herbal methionine supplementation [57,58]. Nevertheless, the inclusion of herbal methionine during the last third of gestation did not increase the maternal live weight.

The results support that 1% herbal methionine supplementation during the last third of gestation positively affects the weight and BMI at birth of the progeny. It is possible that the increased weight and BMI at birth in the T-MET progeny could have been related to the positive impact that methionine plays in fetal tissue growth and development [29,30,33,59]. Similarly, gestational methionine supplementation has been reported to increase birth weight in piglets [32], lambs [34,57], and calves [35,60]. As we mentioned before, we set our nutritional diet to reflect the real-world situation for goats in arid and semiarid regions of Mexico and elsewhere, we observed that the maternal live weight change during the supplementation period was not above the increased fetal weight, implying that the dam utilized its body reserves to support fetal growth and the onset of lactation. Therefore, the birth weight recorded in the present experiment was below the reported for the Alpine breed under intensive conditions [61]; but below or similar to in extensive conditions [52,53,62].

The increased weight and BMI at birth from the T-MET progeny led to faster postnatal development in comparison to T-CTL progeny, respectively. Positive relationships between birth weight and daily live weight gain and between birth weight and weaning weight have been previously reported, indicating that heavier progeny at birth grow faster than lighter progeny [63,64]. Methionine supplementation has been reported to improve the mitochondrial metabolism and the immune function in the newborns [36,65,66], leading to an improved postnatal development [35,67]. Whether the preweaning growth differences between treatments would lead to faster post-weaning development, enhanced metabolic function, the onset of puberty, and, consequently, better reproductive efficiency during their first pregnancy is yet to be determined.

The impact of birth type (singleton vs. multiples) and sex of the progeny (male vs. female) on postnatal growth and development has extensively been reported [2,24,44,49,52,64,68], and our results extend these previous reports. We observed that male kids were heavier and had larger BMI at birth and afterward postnatal development than female kids. The sexual dimorphism observed in these productive variables is due to the differences in the secretory capacity of the somatotropic axis between males and females [69,70]. We also observed that single-born kids were heavier than twin-born kids; heavier offspring stimulate milk production by removing more milk from their dams than lighter lambs [71]. This relationship is positively related to the growth performance of the offspring, thus explaining the differences in growth between single- and twin-born kids [62,68,71].

During lactation, the gestational methionine supplementation did not increase the maternal live weight changes in comparison to those goats that did not receive methionine. Similar findings were reported in goats [72], sheep [56], and cows [38], where similar live weights during lactation was observed between animals receiving methionine in the diet or not. Two hypotheses can be drawn from this observation: firstly, as we mentioned before, it would be possible that the nutrient distribution was to set up the onset of lactogenesis and support fetal growth rather than positively impact the lipid metabolism that would result in maternal live weight gain [73]. Thus, no carryover effect of gestational methionine intake during early lactation occurred. Secondly, during lactation, milk production causes a catabolic state and increased demand for methylated compounds to be used for milk synthesis [74,75]. Dairy animals are able to mobilize body proteins to counteract amino acid deficiencies resulting in maintaining or losing live weight [76,77].

On the other hand, the literature reporting the impact of methionine supplementation on milk yield and milk composition is inconsistent [78]. We observed that methionine supplementation during late gestation did not increase milk yield. Similar findings to our results, indicating that milk yield was not improved after receiving a methionine supplementation were reported by different authors [38,79,80]. However, other studies indicate that supplementation with methionine significantly increased milk yields [81,82,83,84,85]. The discrepancy between our results and others is perhaps the timing of the methionine supplementation. For instance, Titi [85] in Shami goats and Flores et al. [72] in Saanen goats reported a positive impact of methionine supplementation on milk yield and milk protein; however, those authors offered the methionine supplementation during lactation.

Similarly, we observed that the milk composition (protein, lactose, fat, and total solids) was not improved after receiving methionine supplementation during late gestation. However, the reports on milk fat contents are inconsistent [78,80,84], and contrary to our observations, some previous studies reported a positive impact of methionine supplementation on the milk protein content [78,80,84,85,86]. It is possible to hypothesize that methionine does not have a carryover effect and the increased efficiency observed during gestation was no longer available to the mammary gland for milk yield and milk component synthesis. A time effect was observed in the milk composition across the experiment which could have been influenced by the lactation curve, as a negative genetic correlation between milk yield and milk constituents has been reported [87,88]. Nevertheless, methionine supplementation during the last third of gestation did not positively impact the milk yield or milk components of Alpine goats. More research is warranted to extend the supplementation to lactation.

## 5. Conclusions

We concluded that the inclusion of methionine in the maternal diet during the last third of gestation did not increase maternal daily live weight but increases the birth weight and BMI and improved the postnatal growth patterns of the progeny in goats. These effects were independent of changes in milk yields and composition. An increase in birth weight would be expected to increase neonatal survival up to weaning. Relative fatness is required for reproductive success; thus, offspring that are born heavier and bigger can display a faster postnatal growth and accelerate the onset of puberty and increase reproductive success.

## Figures and Tables

**Table 1:** Ingredients and chemical composition of the experimental diet (DM basis). Experimental diet was provided during the last third of gestation.

Experimental Diet			
Ingredient Composition (% in Diet)		Chemical Composition	
Alfalfa hay	24.2	Dry Matter (%)	72.3
Oats hay	45.5	Crude Protein (%)	8.9
Maize silage	30.3	Metabolizable Energy (Mcal/kg)	2.2
		Calcium (%)	0.35
		Phosphorus (%)	0.23

**Table 2:** Effect of supplementation of herbal methionine supplement (Optimethionine^®^) at 1% dry matter based (T-MET) or not (T-CTL) in multiparous Alpine goats during the last third of gestation on maternal live weight at the start and end, live weight change, total birth weight, fetal growth, and total maternal gain. Values are means ± SEM.

Variable	Treatment		*p*-Value
	T-CTL	T-MET	
*n*	30	30	
Enrolment weight (kg)	50.5 ± 1.1	50.3 ± 1.3	0.897
Final weight (kg)	54.2 ± 1.3	52.8 ± 0.1.4	0.472
Live weight change (g/d)	73 ± 10	51 ± 7	0.068
Total Birth Weight (kg)	5.4 ± 0.3	5.1 ± 0.3	0.434
Fetal daily gain (g/d)	108 ± 6	102 ± 5	0.434
Total Maternal Gain (g/d)	−31 ± 10	−47 ± 8	0.231

**Table 3:** Effect of herbal methionine supplement (Optimethionine^®^) on progeny weight and growth variables at birth and weaning of progeny from multiparous Alpine goats that received 0% (T-CTL) or 1% (T-MET; dry matter-based) of herbal methionine supplementation during the last third of gestation. Treatment data combined kid sex and birth-type data. Birth-type data combined treatment and kid sex data. Kid sex data combined treatment and birth-type data. Values are means ± SEM.

Variable	Treatment		*p*-Value	Interactions	
	T-CTL	T-MET		TRT*BT	TRT*Sex
*n*	43	48			
Birth weight (kg)	3.1 ± 0.1	3.5 ± 0.1	0.001	0.199	0.913
Daily live weight gain (g d^−2^)	121 ± 6	142 ± 6	0.010	0.729	0.919
Weaning weight (kg)	8.3 ± 0.2	9.3 ± 0.3	0.004	0.979	0.593
BMI at birth (units kg m^−2^)	0.28 ± 0.01	0.30 ± 0.01	0.010	0.160	0.240
BMI at wean (units kg m^−2^)	0.85 ± 0.03	1.00 ± 0.06	0.030	0.862	0.868
			Sex		*p*-value
			Female	Male	
*n*			46	45	
Birth weight (kg)			3.1 ± 0.1	3.5 ± 0.1	0.004
Daily live weight gain (g d^−2^)			122 ± 6	144 ± 6	0.010
Weaning weight (kg)			8.5 ± 0.3	9.2 ± 0.3	0.040
BMI at birth (units kg m^−2^)			0.27 ± 0.01	0.31 ± 0.01	0.001
BMI at wean (units kg m^−2^)			0.90 ± 0.06	0.96 ± 0.04	0.420
			Birth type		
			Singleton	Twins	
*n*			25	66	
Birth weight (kg)			3.7 ± 0.1	3.1 ± 0.1	0.001
Daily live weight gain (g d^−2^)			165 ± 9	120 ± 4	0.001
Weaning weight (kg)			10.3 ± 0.3	8.3 ± 0.2	0.001
BMI at birth (units kg m^−2^)			0.32 ± 0.01	0.28 ± 0.01	0.002
BMI at wean (units kg m^−2^)			1.14 ± 0.11	0.86 ± 0.03	0.001

## Data Availability

None of the data were deposited in an official repository, yet, information can be made available upon request.
